# Clinical Application of the Modified Scleral Tunnel Intraocular Lens Ciliary Sulcus Suture‐Fixation Technique

**DOI:** 10.1155/joph/5043535

**Published:** 2026-06-15

**Authors:** Bingzhen Li, Yiyun Liu, Xuran Long, Hong Qi

**Affiliations:** ^1^ Department of Ophthalmology, Beijing Key Laboratory of Restoration of Damaged Ocular Nerve, Peking University Third Hospital, Beijing, 100191, China, puh3.net.cn

**Keywords:** complex cataract, intraocular lens suspension, modified scleral tunnel technique, postoperative outcomes, surgical innovation

## Abstract

**Purpose:**

To evaluate the visual outcomes, intraocular lens (IOL) positional stability, and impact on the ocular surface following a novel modified scleral tunnel IOL ciliary sulcus suture‐fixation technique in eyes without adequate capsular support.

**Design:**

A prospective single‐arm cohort study.

**Methods:**

A total of 48 eyes underwent the modified fixation procedure, which utilized a microcurved needle with 10‐0 polypropylene suture to create a scleral tunnel for suture burial. Main outcome measures were assessed preoperatively and at 1 and 3 months postoperatively. Visual function was evaluated by measuring uncorrected distance visual acuity (UDVA) and best‐corrected distance visual acuity (BCVA) using standard logarithmic charts and by manifest refraction. IOL position (decentration and tilt) was quantified using anterior segment optical coherence tomography (AS‐OCT) with ImageJ software analysis. Ocular surface impact was assessed via tear film break‐up time (BUT), corneal fluorescein staining (FL), and the Schirmer I test (SIT).

**Results:**

Visual outcomes significantly improved: postoperative UDVA was 0.41 ± 0.36 logMAR, and BCVA was 0.25 ± 0.32 logMAR, compared to preoperative 1.16 ± 0.65 and 0.61 ± 0.60 logMAR, respectively (both *p* < 0.001). IOL position was stable, with a mean horizontal and vertical decentration of 0.38 mm and 0.39 mm, respectively, and a mean tilt of 3.34° and 3.42°. Ocular surface parameters showed a transient disturbance at 1 month (BUT decreased, FL increased) but returned to near‐baseline levels by 3 months postoperatively.

**Conclusion:**

The modified scleral tunnel IOL fixation technique effectively restores visual function, provides excellent and stable IOL positioning, and induces only transient, reversible disturbance to the ocular surface. This method represents a safe and effective surgical option for eyes lacking capsular support.

## 1. Introduction

Intraocular lens (IOL) suspension is a surgical technique that involves securing an IOL inside the eye—typically to the sclera or iris—using sutures, specialized haptics, or other fixation devices (e.g., intrascleral fixation), without relying on intact capsular bag support. This method is indicated in cases of capsular bag rupture or absence (e.g., trauma and surgical complications), lens dislocation (e.g., Marfan syndrome), and congenital lens abnormalities [1].

In the current landscape of IOL implantation techniques, methods such as scleral suture fixation (classic suspension surgery), sutureless scleral interlayer fixation (e.g., the Yamane technique), iris fixation, and anterior chamber IOL implantation are widely used [2, 3]. However, these traditional methods all have certain limitations. For instance, scleral suture fixation is prone to suture exposure, which may lead to suture degradation or loosening over time, resulting in IOL decentration [4]. Iris fixation can cause pigment dispersion syndrome and glaucoma due to iris chafing [5]. Anterior chamber IOL implantation, on the other hand, carries the risk of corneal endothelial damage.

In light of these issues, this study innovatively employs an improved suspension method—the modified scleral tunnel IOL ciliary sulcus suture‐fixation technique. This method is a safe, effective, and minimally invasive surgical approach that reduces ocular surface trauma by eliminating the need for scleral flap creation, shortening operative time, eliminating the risk of suture knot erosion or exposure, and promoting faster postoperative recovery for patients. The core objective of this study is to systematically evaluate the safety and efficacy of the improved method, aiming to provide a more optimized IOL implantation technique for clinical practice. The safety indicators encompass intraocular pressure (IOP), slit‐lamp examination, other routine tests, and dry eye indicators, while the efficacy indicators include visual acuity and IOL position.

## 2. Research Methods

### 2.1. Study Subjects

This study was a prospective single‐arm cohort study. The study enrolled patients who underwent IOL ciliary sulcus fixation at Peking University Third Hospital between May 2023 and September 2024, with preoperative diagnoses including IOL dislocation, posttraumatic aphakia, anterior chamber IOL, and lens dislocation. All patients underwent comprehensive ophthalmic and systemic evaluations. The study was approved by the Medical Ethics Committee of Peking University Third Hospital (M2023663) and complied with the Declaration of Helsinki. All participants involved in this study provided written informed consent prior to enrollment.

### 2.2. Preoperative Ocular Examinations

All enrolled patients underwent detailed ocular and systemic assessments. Routine preoperative examinations included the following: uncorrected distance visual acuity (UDVA), IOP measurement, slit‐lamp examination, manifest refraction, BCVA, biometry, and corneal topography. These tests aimed to confirm eligibility and rule out coexisting systemic or ocular diseases.

We also collected baseline information from the patients, including gender, age, eye side, etiology, and preoperative diagnosis.

#### 2.2.1. Routine Examination

We conducted comprehensive preoperative assessments, including visual acuity testing, IOP measurement, and refraction examination. Visual acuity was evaluated using standard logarithmic visual acuity charts to measure UDVA and BCVA. All visual acuity measurements were performed by the same experienced optometrist to ensure consistency. IOP was measured using noncontact tonometry (NCT; Topcon CT‐800, Tokyo, Japan). Refraction assessment included both objective and manifest refraction to determine the BCVA. Objective refraction was performed automatically using a fully automated autorefractor (Topcon Corporation, Japan), while manifest refraction was conducted by an optometrist using a phoropter (Rodenstock, Germany). Throughout the process, we implemented strict quality control measures to ensure the accuracy and reliability of all measurements.

#### 2.2.2. Ocular Biological Measurement

Ocular biometric parameters were primarily used for calculating the IOL spherical power, with key measurements including axial length (AL), anterior chamber depth (ACD), corneal curvature, lens thickness (LT), and white‐to‐white (WTW) distance. All parameters were measured using the IOLMaster 700 (Carl Zeiss Meditec AG, Jena, Germany). The examiner was required to verify the corresponding measurement modes to ensure that the acquired images met quality standards.

#### 2.2.3. Postoperative Measurement of IOL Morphology and Position Parameters

For this study, postoperative measurements of IOL decentration (mm) and tilt (°) were performed using anterior segment optical coherence tomography (AS‐OCT) with the CASIA2 system (Tomey Corporation, Nagoya, Japan) and analyzed using ImageJ software (Version 1.8.0, National Institutes of Health, USA).

The CASIA2 AS‐OCT system utilizes a 1310‐nm wavelength light source to acquire 128 cross‐sectional images within seconds, generating three‐dimensional reconstructions of the anterior segment with excellent reproducibility and consistency, which is ideal for assessing IOL position.

The acquired AS‐OCT images were processed and analyzed with ImageJ software. A best‐fit algorithm was applied to identify circles corresponding to the IOL’s optical surfaces. This process allowed for the determination of the IOL’s optical center and axis. These values were then compared with the pupillary or visual axis to calculate the exact magnitude of IOL decentration and degree of tilt.

#### 2.2.4. Additional Preoperative Examinations

All study eyes underwent a standardized preoperative evaluation protocol including ocular B‐scan ultrasonography, posterior segment optical coherence tomography, and color fundus imaging to comprehensively assess vitreoretinal status. Particular attention was given to eyes with confirmed or suspected vitreoretinal pathologies.

Corneal endothelial evaluation was performed using the EM3000 specular microscope (Tomey Corporation, Nagoya, Japan) to quantify corneal endothelial cell density and determine the percentage of hexagonal cells. All diagnostic findings were systematically reviewed by the operating surgeon to identify any ocular comorbidities that might contraindicate IOL implantation.

### 2.3. Intraoperative

**FIGURE 1:**
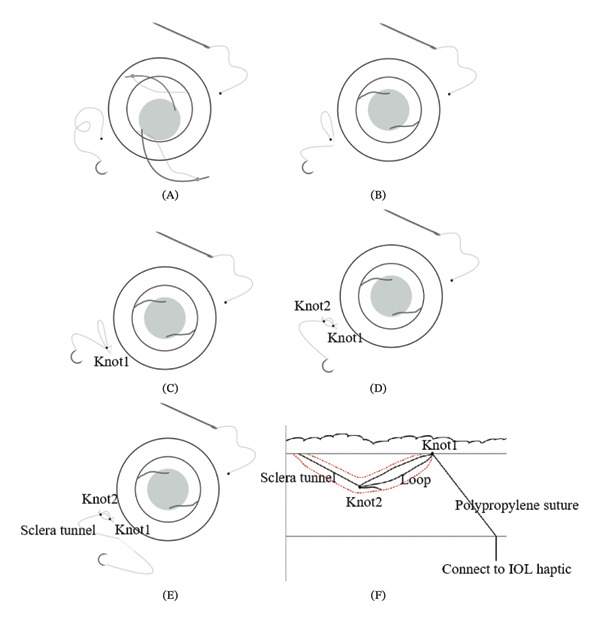
Schematic diagram of the IOL scleral suturing procedure. (A): Tie and fix the sutures from the 2:00 and 8:00 positions to the distal end of the IOL haptics, and then adjust the IOL haptics to their intended positions. (B): Using a curved needle with 10‐0 polypropylene suture at the 2:00 position, pass it through the lamellar sclera 2 mm from the limbus to create a suture loop. (C): Tie the polypropylene suture (with the curved needle) to the suture loop (Knot 1) to secure the IOL. (D): Make another knot (Knot 2) 3–4 mm distal to Knot 1, trim the excess suture as short as possible, and form an “O”‐shaped double knot loop. (E): Near the base of Knot 1, use a curved needle with 10‐0 polypropylene suture to create a scleral tunnel (5–8 mm in length) within the lamellar sclera, exiting at the distal end. (F): Bury the “O”‐shaped double knot loop into the tunnel, and then trim the suture flush at the exit point to prevent suture exposure.

For patients with cataract or lens dislocation, lensectomy was performed first following IOL fixation. All surgical procedures were performed by a single experienced ophthalmologist to eliminate interoperator variability, and the IOL implantation is illustrated in Figure 1.

Finally, the viscoelastic material in the anterior chamber was completely aspirated, and the incision was checked for self‐sealing (typically requiring no sutures).

### 2.4. Postoperative

#### 2.4.1. Routine Examination

All patients were required to return for outpatient follow‐up visits at 1 month and 3 months postoperatively. At the 1‐month follow‐up, routine examinations included distance visual acuity, IOP, manifest refraction, and slit‐lamp examination. The methods for measuring distance visual acuity, IOP, and manifest refraction were the same as those used preoperatively. The slit‐lamp examination focused on the healing of the surgical incision, corneal edema, anterior chamber inflammation, and IOL position. These assessments were conducted to ensure surgical safety and to document any complications.

#### 2.4.2. IOL Position

**FIGURE 2:**
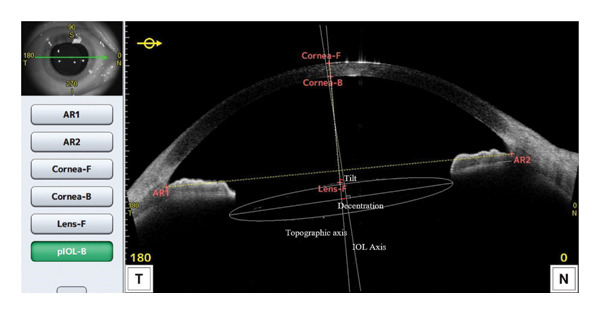
Tilt and decentration measurement of one patient as an example.

AS‐OCT examinations were performed (postoperative cataract mode) by an experienced technician at 1 month after surgery for all patients. The obtained 2D images were analyzed with Image *J* software (Version 1.8.0). The IOL axis, which passes through the optical center of the IOL, was determined by plotting the best‐fit circles for the anterior and posterior IOL surfaces. The corneal topographic axis was defined as the line perpendicular to the corneal vertex. The IOL decentration was measured as the distance between these two axes, and the IOL tilt was measured as the angle between them (Figure 2, written informed consent for the publication of the image was obtained from the relevant patient. All identifiable personal information has been fully anonymized in the figure).

#### 2.4.3. Dry Eye Assessment

Additional specialized testing evaluated dry eye parameters through tear break‐up time (BUT) assessment, fluorescein staining (FL) patterns, and the Schirmer I test (SIT) measurements [6].

BUT: The purpose of the BUT test is to assess the stability of the tear film and reflect whether the ocular surface lubrication function is impaired. The patient looks forward, and the tear film is observed under cobalt blue light with a slit lamp. Sodium FL is instilled into the conjunctival sac (or a fluorescein test strip can be used). The patient is instructed to blink naturally and then keep the eyes open, and the time (in seconds) until the first dry spot appears on the corneal surface is recorded. A result of ≥ 10 s is considered normal, 5–9 s indicates mild dry eye, and < 5 s indicates moderate to severe dry eye.

FL: The purpose of the FL test is to assess the degree of corneal epithelial damage and locate punctate lesions related to dry eye. Sodium fluorescein is instilled into the conjunctival sac and gently rinsed with saline. The corneal staining is observed under cobalt blue light and recorded according to a scoring system. Commonly used scoring standards include the Oxford grading or the NEI scale. Oxford grading (0–5 grades): Grade 0: no staining; Grade 1: a few punctate stains (< 5 points); Grade 3: moderate scattered staining; Grade 5: fusion or extensive staining. NEI scale: The cornea is divided into 5 zones, with each zone scored from 0 to 3 points (total score 15 points).

SIT: The purpose of the SIT is to assess the basal tear secretion and differentiate aqueous tear deficiency dry eye. In the unanesthetized state (the Schirmer I test), a test strip is placed in the outer 1/3 of the lower conjunctival sac. The patient is instructed to close the eyes gently, and after 5 min, the strip is removed, and the wetting length (in mm) is measured. Normal value: ≥ 10 mm/5 min; mild reduction: 5–9 mm; severe reduction: < 5 mm.

During follow‐up, the symptoms and signs of the patient should be taken into account to promptly determine whether there are any postoperative complications. For patients who develop complications, the conditions should be documented in detail, and corresponding clinical management measures should be taken. The decision to exclude the patient from the study group should be based on the type and severity of the complications.

### 2.5. Statistical Analysis

During data analysis, visual acuity results were converted from decimal notation to logMAR visual acuity for further analysis based on the conversion relationship between the two. For example, a decimal visual acuity of 0.5 was converted to a logMAR visual acuity of log(1/0.5) = 0.30. The spherical equivalent was calculated as the sphere power plus half of the cylinder power, using the following formula: spherical equivalent (SE) = sphere (S) + 1/2 × cylinder (C).

Statistical analysis was performed using SPSS 29.0 software (IBM Corporation, Armonk, NY, USA), and graphical illustrations were created using GraphPad Prism 9.5 software (GraphPad Software, Inc., San Diego, CA, USA). The data were confirmed to follow a normal distribution. For categorical variables, descriptive statistics were employed to present the data in terms of counts and proportions.

When comparing continuous data between preoperative and 3‐month postoperative time points, paired sample *t*‐tests were used. For comparisons involving three time points (preoperative, 1 month postoperative, and 3 months postoperative) for BUT, FL, and SIT, repeated‐measures analysis of variance (ANOVA) was applied. Descriptive statistics were used to report the mean and range of measurements such as decentration (mm) and tilt angle (°). A *p* value of less than 0.05 was considered statistically significant.

## 3. Results

### 3.1. Baseline

As shown in Table 1, this study included 48 patients (48 eyes), with 34 males (70.83%) and 14 females (29.17%). The right eye was affected in 27 cases (56.25%) and the left eye in 21 cases (43.75%). Preoperative diagnoses were as follows: IOL‐capsular bag complex dislocation (11 eyes, 22.92%), IOL dislocation (13 eyes, 27.08%), posttraumatic aphakia (9 eyes, 18.75%), anterior chamber IOL (2 eyes, 4.17%), and lens dislocation/subluxation (13 eyes, 27.08%). The mean age was 58.73 ± 13.68 years (range: 17–88 years), with follow‐up from 3 to 16 months. The average surgical time was 30.35 ± 10.21 min. IOLs used were AR40e (38 cases) (Abbott Medical Optics, Santa Ana, CA, USA), 839MP (2 cases) (Carl Zeiss Meditec AG, Jena, Germany), PY60‐AD (6 cases) Hoya Surgical Optics, Tokyo, Japan), and Hoya PC‐60R (2 cases) (Hoya Surgical Optics, Tokyo, Japan).

**TABLE 1:** Preoperative baseline data of 48 patients (48 eyes) undergoing ciliary sulcus suture fixation of IOL with the scleral tunnel technique.

Baseline characteristics	Number of cases (%)
Gender	
Male	70.83
Female	29.17
Eye	
Right eye	56.25
Left eye	43.75
Preoperative diagnosis	
IOL capsular complex dislocation	22.92
IOL dislocation	27.08
Posttraumatic aphakia	18.75
Anterior chamber IOL	4.17
Lens dislocation/subluxation	27.08

*Note:* IOL = intraocular lens.

### 3.2. Preoperative Visual Acuity, IOP, and Refractive Outcomes

Preoperative and postoperative parameters are summarized in Table 2. Preoperative examinations revealed a UDVA of 1.16 ± 0.65 logMAR, BCVA of 0.61 ± 0.60 logMAR, spherical equivalent of 2.63 ± 6.19 D, astigmatism of −2.19 ± 1.64 D, and IOP of 17.85 ± 8.08 mmHg. At 3 months postoperatively, significant improvements were observed: UDVA improved to 0.41 ± 0.36 logMAR (*p* < 0.001), BCVA to 0.25 ± 0.32 logMAR (*p* < 0.001), spherical equivalent reduced to −1.18 ± 1.48 D (*p* < 0.001), and astigmatism decreased to −0.77 ± 0.45 D (*p* < 0.001). IOP measured 15.96 ± 3.95 mmHg showed no statistically significant change (*p* = 0.119).

**TABLE 2:** The visual acuity, intraocular pressure, and refractive status of 48 patients (48 eyes) before and after IOL ciliary sulcus suture fixation with scleral tunnel (mean ± SD).

Time	UDVA (logMAR)	BCVA (logMAR)	Refractive status		IOP (mmHg)
			SE (D)	Astigmatism (D)	
Preop.	1.16 ± 0.65	0.61 ± 0.60	2.63 ± 6.19	−2.19 ± 1.64	17.85 ± 8.08
3 months	0.41 ± 0.36	0.25 ± 0.32	−1.18 ± 1.48	−0.77 ± 0.45	15.96 ± 3.95
*t* value	10.563	6.597	4.141	5.569	1.587
*p* value	< 0.001	< 0.001	< 0.001	< 0.001	0.119

Abbreviations: BCVA, best‐corrected visual acuity; D, diopter; IOL, intraocular lens; IOP, intraocular pressure; logMAR, logarithm of the minimum angle of resolution; SE, spherical equivalent; UDVA, uncorrected distant visual acuity.

### 3.3. Evaluation of dry Eye Indicators

**FIGURE 3:**
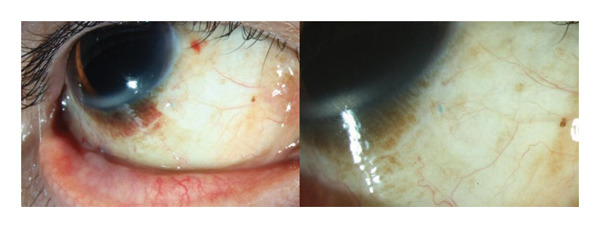
Subconjunctival position of the scleral fixation knots was well tolerated at 1 week and 1 month postsurgery.

The IOL fixation knots remained completely covered by the conjunctiva without exposure at both 1 week and 1 month after surgery (Figure 3, written informed consent for the publication of the image was obtained from the relevant patient. All identifiable personal information has been fully anonymized in the figure). The changes in dry eye indicators are summarized in Table 3. Tear‐film evaluation demonstrated preoperative values of 6.79 ± 1.86 s for BUT, 0.54 ± 0.71 for the corneal FL score, and 9.29 ± 2.00 mm for SIT. At 1 month postoperatively, BUT decreased to 4.58 ± 1.49 s, FL score increased to 1.08 ± 0.85, and SIT decreased to 8.90 ± 1.43 mm. By 3 months, these parameters returned to near baseline levels: BUT 6.73 ± 1.85 s, FL score 0.52 ± 0.62, and SIT 9.33 ± 1.94 mm, with only SIT showing marginal statistical significance (*p* = 0.027).

**TABLE 3:** The BUT, FL scores, and SIT result of 48 patients (48 eyes) before and after IOL ciliary sulcus suture fixation with scleral tunnel (mean ± SD).

Time	BUT (s)	FL (score)	SIT (mm)
Preop.	6.79 ± 1.86	0.54 ± 0.71	9.29 ± 2.00
1 month postop.	4.58 ± 1.49	1.08 ± 0.85	8.90 ± 1.43
3 months postop.	6.73 ± 1.85	0.52 ± 0.62	9.33 ± 1.94
*F* value	89.848	15.050	3.917
*p* value	< 0.001	< 0.001	0.027

Abbreviations: BUT, tear film break‐up time; FL, corneal fluorescein staining; IOL, intraocular lens; SIT, Schirmer I test.

### 3.4. Evaluation of IOL Position

Postoperative IOL positioning analysis showed excellent stability (Table 4), with a horizontal decentration of 0.38 mm (range: 0.02–1.14 mm) and tilt of 3.34° (range: 0.16°–7.60°), while vertical measurements showed decentration of 0.39 mm (range: 0.06–1.02 mm) and tilt of 3.42° (range: 0.36°–8.26°), all within clinically acceptable ranges.

**TABLE 4:** Evaluation of IOL location after surgery.

	Decentration (mm)		Tilt(°)	
	Mean	Range	Mean	Range
Level	0.38	0.02–1.14	3.34	0.16–7.60
Vertical	0.39	0.06–1.02	3.42	0.36–8.26

Abbreviation: IOL, intraocular lens.

Complications included one case of IOL pupillary capture that resolved with mydriasis, one case of cystoid macular edema at 2 weeks postoperatively that resolved within 1 week following 20 mg retrobulbar triamcinolone injection, and one case of minor intraoperative vitreous hemorrhage caused by straight to curved needle passage that completely resolved by 3 weeks. No eyes developed suture exposure, suture breakage, persistent IOP fluctuation, prolonged postoperative inflammation, or infectious endophthalmitis.

## 4. Discussion

We have developed a novel method for IOL suspension using a modified scleral tunnel technique with 10‐0 polypropylene suture. This method provides a new solution for IOL fixation without capsular support, particularly for patients with aphakia and lens dislocation. Its clinical significance lies in reducing surgical trauma and operative time, being applicable to various types of IOLs (thus overcoming the limitations of sutureless techniques), and minimizing the use of consumables to lower surgical costs.

Our innovation lies in directly using a microcurved needle on the suspension suture to create a scleral tunnel. This simplifies the surgical procedure and reduces the average surgical time to 30.35 min. Moreover, our method supports a wide range of IOLs (such as one‐piece, three‐piece, and plate‐haptic IOLs), is highly versatile, and eliminates the need for a microtunnel knife and scleral flap sutures, thereby reducing the use of consumables and lowering costs.

Compared to other methods reported in the literature, our approach offers significant advantages. Currently, the literature reports four main techniques for ciliary sulcus fixation of IOLs:

Scleral flap suture fixation: This method involves creating a triangular or rectangular scleral flap, puncturing the ciliary sulcus, guiding a polypropylene suture with a long needle to fixate the IOL optic or haptic, securing both ends of the suture to the scleral bed, and finally covering the scleral flap and suturing the conjunctiva [7, 8]. However, it has notable drawbacks: the procedure is complex, requiring the creation and suturing of a scleral flap and conjunctival incision, leading to greater ocular surface trauma; there is a high risk of suture exposure [9]; and it necessitates the use of microtunnel knives and 10‐0 sutures, increasing material costs.

Yamane double‐needle sutureless fixation: This technique constructs an intrascleral tunnel 2 mm from the limbus using a 30‐G needle, inserts the straight haptics of a three‐piece IOL (e.g., CT Lucia model) into the needle, and thermally shapes the haptic ends into a “mushroom head” structure for anchorage. Its limitations include applicability only to specific three‐piece IOLs (requiring straight and rigid haptics) [4, 10] and the risk of haptic displacement or breakage during thermal shaping, which may lead to IOL tilt 1 [11].

Flanged technique (modified Yamane method): A 27‐G needle is used to puncture the sclera, followed by fixation of the IOL haptics with 5‐0/6‐0 polypropylene sutures. The suture ends are melted to form flange structures that retract into the sclera for anchorage. Key disadvantages include the significantly higher risk of intraoperative bleeding and choroidal detachment due to the thicker 27‐G needle and 5‐0/6‐0 sutures [12], as well as the need for four scleral punctures—doubling the bleeding risk compared to traditional two‐puncture methods.

Modified suture fixation (Jin Haiying technique): This approach is a modification of the classic technique that eliminates the need for a scleral flap and microtunnel knife. It instead uses a 30‐gauge needle and 8‐0 polypropylene sutures for IOL fixation. However, the thicker 8‐0 suture increases the risk of bleeding and tissue trauma during transscleral passage. In comparison, our method offers several advantages: First of all, our technique utilizes a microbent needle to directly construct the tunnel as a surgical approach, eliminating the need for scleral flap creation (as required in scleral flap fixation and the Jin Haiying technique), thereby reducing surgical time and minimizing ocular surface trauma.

Regarding suture selection, compared to the 5‐0/6‐0 sutures used in the flange technique and the 8‐0 sutures in the Jin Haiying method, our approach employs 10‐0 sutures, further reducing the risk of intraoperative bleeding.

Concerning applicability, in contrast to the Yamane technique which is only suitable for specific three‐piece IOLs, our method can be applied to a wider range of cases, including IOL‐capsular bag complex dislocation, suture fixation of one‐piece IOLs, and dislocations involving loop or plate‐haptic IOLs.

With respect to material costs, unlike scleral flap fixation and the Jin Haiying technique, our method eliminates the need for microtunnel knives or 10‐0 sutures for scleral flap and conjunctival closure, significantly reducing material expenses.

In terms of safety, the surgical complication rate was low, with only three minor complications (one case of IOL pupillary capture, one case of macular edema, and one case of vitreous hemorrhage), none of which required secondary surgical intervention. There were no severe complications such as endophthalmitis or suture exposure. In terms of efficacy, 3 months postoperatively, UDVA improved significantly from 1.16 ± 0.65 logMAR to 0.41 ± 0.36 logMAR (*p* < 0.001). The IOL decentration (0.38 mm horizontally and 0.39 mm vertically) and tilt angle (3.34°–3.42°) were stable. The tear film BUT decreased 1 month postoperatively but returned to preoperative levels by 3 months. Although transient dry eye occurred, it indicated that surgical trauma was controllable.

This modified scleral tunnel technique simplifies surgery and reduces trauma. IOLs were fixated in the ciliary sulcus, which provides a physiological position for optimal effective lens position and visual outcomes, while reducing deep intraocular manipulation (such as pars plana fixation) and complications. The 10‐0 polypropylene suture and microcurved needle minimized tissue irritation. No uveitis, glaucoma, hyphema (UGH syndrome), or iris pigment dispersion was observed, verifying the safety of ciliary sulcus fixation.

Although among the included IOLs, only the AT LISA tri 839MP is a one‐piece plate‐haptic trifocal IOL, the 2 cases with 839MP implantation showed comparable IOL stability and visual outcomes with other IOL models. The 839MP trifocal IOL has high requirements for positional stability to ensure optical quality (tilt > 5° or decentration > 0.5 mm may affect optical quality). Our AS‐OCT results showed its positional stability (decentration < 0.4 mm, tilt < 3.5°), with no related complications. Combined with relevant studies [13], scleral suture fixation for trifocal IOL is feasible. It indicated that this scleral fixation method is applicable to both 3‐piece monofocal IOLs and the 839MP trifocal IOL. With the increasing clinical application of 839MP, it is inevitable to encounter cases of IOL dislocation, and this scleral fixation method provides a reliable and stable option for patients who refuse IOL replacement, ensuring the stability of IOL position and maintaining the expected visual effect.

Several limitations should be acknowledged. Postoperative keratometry was not routinely performed in this study. Future studies may add postoperative corneal astigmatism measurements and compare them with refractive astigmatism, which can help further verify IOL position and evaluate the optical impact caused by IOL positional stability. In addition, this study is a prospective single‐arm cohort study without a control group. Therefore, direct comparisons of surgical efficiency and recovery speed with conventional techniques (scleral flap fixation and Yamane technique) are not available, and comparative studies will be conducted in future research.

## 5. Conclusion

The modified scleral tunnel IOL ciliary sulcus suture‐fixation technique offers significant advantages, including reduced surgical trauma, versatility with various IOL types, and no suture exposure observed in this series. Its simplicity, cost‐effectiveness, and excellent postoperative outcomes support its clinical adoption for patients lacking capsular support. Further validation with larger studies is recommended to confirm its long‐term benefits.

## Author Contributions

Bingzhen Li and Yiyun Liu (cofirst authors, equal contribution) are responsible for study design, data analysis, and the manuscript writing. Xuran Long is responsible for data collection. Hong Qi is responsible for supervising the research, data interpretation, critical revision of the manuscript, and guiding the study design.

## Funding

This study was supported by the National Natural Science Foundation of China (No. 82371026).

## Disclosure

The funding organizations had no role in the design or conduct of this research.

All authors have no financial or proprietary interest in the materials presented herein. All authors attest that they meet the current ICMJE criteria for authorship.

## Conflicts of Interest

The authors declare no conflicts of interest.

## Data Availability

The datasets generated during and/or analyzed during the current study are available from the corresponding author upon reasonable request.
